# Sarcoidosis and Systemic Sclerosis: Strange Bedfellows

**DOI:** 10.1155/2017/7851652

**Published:** 2017-08-24

**Authors:** Micah Yu, Vaneet K. Sandhu, Sheila D. Lezcano, Kanwaljeet Maken, Shannon Kirk, Karina D. Torralba

**Affiliations:** ^1^Department of Medicine, Loma Linda University, Loma Linda, CA, USA; ^2^Division of Rheumatology, Department of Medicine, Loma Linda University, Loma Linda, CA, USA; ^3^Division of Pulmonary and Critical Care, Department of Medicine, Loma Linda University, Loma Linda, CA, USA; ^4^Department of Radiology, Loma Linda University, Loma Linda, CA, USA

## Abstract

Coexistence of systemic sclerosis and sarcoidosis is rare. Both have predominant lung manifestations, each with distinctive features on computed tomography (CT) of the chest. We present herein a 52-year-old male with limited systemic sclerosis manifested primarily by sclerodactyly and subsequently by shortness of breath. A series of CT scans of the chest were reviewed. Initial CT chest one year prior to sclerodactyly onset revealed bilateral hilar and right paratracheal, prevascular, and subcarinal adenopathy. Five-year follow-up demonstrated thin-walled cysts, mediastinal lymphadenopathy, and nonspecific nodules. Due to progression of dyspnea, follow-up CT chest after one year again demonstrated multiple cysts with peripheral nodularity and subpleural nodules, but no longer with hilar or mediastinal adenopathy. Diagnostic open lung biopsy was significant for noncaseating granulomas suggestive of sarcoidosis. This is the first known case of a patient with systemic sclerosis diagnosed with sarcoidosis through lung biopsy without radiographic evidence of hilar or mediastinal lymphadenopathy at the time of biopsy. A review of cases of concomitant sarcoidosis and systemic sclerosis is discussed, including the pathophysiology of each disease with shared pathways leading to the development of both conditions in one patient.

## 1. Introduction

Sarcoidosis is a multisystem disease of unknown etiology defined by noncaseating granulomas that may be seen on lung imaging as bilateral hilar lymphadenopathy and/or interstitial lung disease (ILD) [1, 2]. The association of sarcoidosis with other autoimmune diseases may include systemic sclerosis, Hashimoto's thyroiditis, systemic lupus erythematosus, rheumatoid arthritis, and more [3, 4]. In contrast to findings of mid- to upper-lobe interstitial lung disease (ILD) with hilar lymphadenopathy in sarcoidosis, the most common lung manifestation of systemic sclerosis is lower-lobe ILD, which is typically characterized by a nonspecific interstitial pneumonia pattern (groundglass opaci-ties, pulmonary fibrosis, and honeycomb cysts) [5–7]. We report herein the atypical case of a Caucasian male with systemic sclerosis who was eventually diagnosed with pulmonary sarcoidosis. The pathophysiology of each disease, including shared pathways leading to the development of both conditions, is reviewed in addition to previous reports of patients with concomitant systemic sclerosis and sarcoidosis.

## 2. Case Report

A 52-year-old Caucasian male presented initially to the Loma Linda University Rheumatology Clinic in 2007 with swelling in the hand and foot joints. He also endorsed a history of dyspnea on exertion; however, due to lack of follow-up after the initial visit, no diagnosis or treatment was provided at the time. Incidental review of chest CT done one year previously through his primary care provider was significant for bilateral hilar and right paratracheal, prevascular, and subcarinal adenopathy. Five years later, the patient presented again to the rheumatology clinic with Raynaud's phenomenon, fingertip ulcerations, skin thickening and tightness over his fingers, and hypo- and hyperpigmented patches over the lower extremities. He also endorsed shortness of breath but denied mucocutaneous lesions, gastrointestinal manifestations, hair loss, or sicca symptoms. Physical examination confirmed sclerodactyly, fingertip ulcerations, and intermixed hypo- and hyperpigmented skin changes. Despite a normal cardiopulmonary physical examination, chest X-ray implicated interstitial pulmonary fibrosis with nodules noted both in the right upper lobe (5.1 mm) and in the left lower lobe (6.6 mm). Follow-up imaging by high-resolution chest CT (HRCT) confirmed multiple nonspecific nodules in the right upper and middle lobes and bilateral oblique fissures ranging in size from 5 to 9 mm. This was in addition to mediastinal lymphadenopathy and multiple thin-walled air cysts bilaterally with peripheral intralobular groundglass opacities suggestive of lymphocytic interstitial pneumonia (LIP).

On laboratory evaluation, serum antinuclear antibody (1 : 640 centromere pattern, 1 : 160 speckled) and rheumatoid factor (RF), anti-SSA, anti-SSB, and anti-centromere antibodies were positive. C-reactive protein and erythrocyte sedimentation rate were normal. Antibodies to double-stranded DNA (dsDNA), Smith, Scl-70, U1RNP, and cyclic-citrullinated peptide (CCP) were negative. Urinalysis, comprehensive metabolic profile, and angiotensin-converting enzyme (ACE) were normal. Infectious evaluation, including quantiferon testing and histoplasma and coccidioides serologies, was negative. Pulmonary function testing (PFT) revealed an FEV1/FVC ratio of 98% with diffusion capacity of carbon monoxide (DLCO) 71%. This combination of clinical and serologic features yielded a diagnosis of limited systemic sclerosis with interstitial lung disease (ILD).

One year later, the patient endorsed progression of exertional dyspnea in addition to a new onset dry cough. Transthoracic echocardiography revealed a normal ejection fraction (65%) without valvular abnormality or pulmonary hypertension. Follow-up HRCT demonstrated multiple cysts bilaterally with peripheral nodularity and small subpleural nodules. Interestingly, previously noted hilar and mediastinal adenopathy were not present. PFT disclosed likely progression of fibrosis with an FEV1/FVC ratio of 96% and worsened DLCO 63%. Lung biopsies were obtained by way of video-assisted thoracoscopy with right lung wedge resection and mediastinal lymph node dissection. The histologic findings unexpectedly revealed prominent nonnecrotizing granulomatous inflammation of the parenchyma, pleura, and lymph nodes, all of which supported a diagnosis of sarcoidosis.

In this long-term follow-up of a patient initially diagnosed with systemic sclerosis, the subsequent clinical, laboratory, and histologic evaluation yielded an ultimate diagnosis of an overlap syndrome of systemic sclerosis and sarcoidosis with ILD. For this, treatment was initiated with mycophenolate mofetil 500 mg twice daily by mouth in addition to diltiazem 90 mg every 12 hours by mouth for Raynaud's phenomenon. During follow-up visits thus far, the patient has demonstrated decreased shortness of breath and cough, with objective follow-up (i.e., PFT, 6MWT) examination still pending. Further longitudinal follow-up with the patient is anticipated in addition to reviewing response to therapy by way of follow-up imaging.

## 3. Discussion

Sarcoidosis and systemic sclerosis are both multisystem disorders of unknown etiology. While sarcoidosis manifests as a noncaseating granulomatous disease affecting most commonly the lungs, skin, and eyes, systemic sclerosis is a connective tissue disorder characterized by cutaneous sclerosis, visceral fibrosis, and vasculopathy. Because the incidence of sarcoidosis is 50 to 400 per million per year and the incidence of systemic sclerosis is 20 per million per year, the likelihood of overlap disease is expected to be low [8]. Other rarely reported autoimmune diseases that have been reported in association with sarcoidosis include rheumatoid arthritis, systemic lupus erythematosus, Sjögren syndrome, and thyroid disease [3, 9].

Enzenauer and West have demonstrated that, of 569 consecutive patients with connective tissue disease, six patients (1%) developed concomitant sarcoidosis over a 10-year period [3]. More specific to the case at hand, while rare, the coexistence of sarcoidosis with systemic sclerosis has been reported in the literature. Of twenty-nine published cases demonstrating an association of systemic sclerosis with sarcoidosis, only 16 were fully accessible. A review of these cases revealed a predominance of females (74%) with an average age of 54 years [2, 3, 10–21]. Histologic evidence by biopsy of the lung was noted in 8 subjects, biopsy of the skin in 6 subjects, and biopsy of the muscle in 1 subject. Most patients with serum Scl-70 antibody were male (78%), while anti-centromere antibodies were typically demonstrated among females (88%), implicating a relationship between autoantibody profiles and gender in the concurrence of these diseases [22].

Sarcoidosis and systemic sclerosis independently demonstrate lung pathology manifested clinically by dyspnea and cough and radiographically by distinct features on CT of the chest. Pulmonary disease in systemic sclerosis is characterized by ILD and/or pulmonary hypertension, both of which are significant causes of morbidity and mortality that necessitate frequent screening. Systemic sclerosis-ILD is typically a nonspecific interstitial pneumonia (NSIP) pattern with groundglass opacities, interstitial pulmonary fibrosis, and honeycomb cystic changes in the lower lung zones [5, 6, 23]. In fact, NSIP is the most common histopathologic diagnosis in systemic sclerosis–related lung disease patients who underwent surgical lung biopsy [6]. In contrast, lung manifestations of sarcoidosis include bilateral hilar and mediastinal lymphadenopathy but may also involve beading or irregular thickening of the bronchovascular bundles, bronchial wall thickening, nodules along the bronchi, blood vessels, subpleural lesions, and mid- to upper-lobe-predominant groundglass opacities identified on HRCT chest [3, 7, 22] and confirmed by biopsy if clinically indicated.

In the case presented, mediastinal and hilar adenopathy were present on the initial two HRCT scans of the chest but not so on the last HRCT done just prior to lung biopsy. Rather, the last two chest HRCT scans revealed cystic changes more suggestive of LIP, which is a nonspecific finding of inflammatory lung disease [23, 24]. Similar to other HRCT findings of sarcoidosis, LIP may demonstrate thickened bronchovascular bundles, nodules, groundglass opacities, cysts, and thickening of interlobular septa [25].

While the pathogenesis of sarcoidosis and systemic sclerosis remains unclear, both diseases occur as a result of immune system activation with a subsequent inflammatory reaction in some genetically predisposed individuals (i.e., mutations, SNPs, and chromosomal aberrations) and others with environmental exposures [26, 27]. With regard to cellular pathophysiology, sarcoidosis is a T-helper 1- (Th1-) mediated disease that results in noncaseating epithelioid cellular granuloma formation [28]. CD4^+^ T-lymphocytes have been identified in the tissues of patients with sarcoidosis and appear to be most concentrated within the granulomatous lesion [29]. Th1 cytokines, interferon- (IFN-) λ and interleukin- (IL-) 2, are elevated in sarcoidosis, while T-helper 2 (Th2) cytokines, IL-4 and IL-5, are decreased [30]. On the other hand, the complex pathophysiology of systemic sclerosis varies based on the stage of the disease. Early and active stages of systemic sclerosis are a result of the Th2 pathway [31], with progression of the disease believed to result from damage of the endothelial lining in small blood vessel walls leading to impaired circulation and subsequent tissue hypoxia and collagen deposition [32]. While this foundation of systemic sclerosis and sarcoidosis pathophysiology is significantly different, both conditions have demonstrated commonalities with regard to cytokines. IL-1α has been implicated as a producer of IL-6, which is elevated in the serum as well as fibroblasts of patients with systemic sclerosis when compared with healthy individuals [33]. It is also found to be higher in the bronchoalveolar lavage fluid of patients with systemic sclerosis, implicating a role in the pulmonary pathogenesis of systemic sclerosis [33]. Similarly, IL-6 and IL-8 were found to be elevated in the bronchoalveolar lavage fluid of patients with active sarcoidosis [34].

Fibrosis is a pathologic attribute shared by both systemic sclerosis and sarcoidosis, specifically with multisystemic fibrosis in systemic sclerosis and a more localized lung fibrosis in 20–25% of patients with sarcoidosis [35]. Both conditions have demonstrated an increased prostaglandin endoperoxidase-2 (PTGS2) or cyclooxygenase-2 (COX-2) expression. In sarcoidosis, a promoter polymorphism, −765G>C, has demonstrated an association with lung fibrosis onset [36, 37]. The cytokine TGF-β1 induces COX-2 expression through smad signaling pathways and is known to drive fibrosis in systemic sclerosis [36]. Experimental use of a neutralizing antibody against TGF-β1 has demonstrated reversal of cutaneous fibrosis in systemic fibrosis [37, 38]. Levels of TGF-β1 are also increased in peripheral blood lymphocytes and bronchoalveolar cells in sarcoidosis patients with lung involvement [36, 38]. TGF-β2 and TGF-β3 gene polymorphisms have been implicated in cutaneous fibrosis of systemic sclerosis [39] with similar findings demonstrated by Kruit and colleagues in a four-year follow-up of sarcoidosis patients developing pulmonary fibrosis when bearing a genetic variation of TGF-β3 [27].

Chemokines are cytokines that are crucial in leukocyte recruitment to inflamed tissues. In a study conducted by Furuse and colleagues, patients with systemic sclerosis demonstrated greater IL-8 and GRO-α that correlated with a decrease in DLCO compared with their counterparts with SLE and type II diabetes [40]. van Bon and colleagues further established that CXCL4 downregulates the antifibrotic cytokine IFN-γ and upregulates the profibrotic cytokines IL-4 and IL-13 [41]. This was demonstrated in patients with high CXCL4 at baseline compared with other biomarkers for systemic sclerosis who experienced a more rapid decline in DLCO, greater prevalence of HRCT-confirmed lung fibrosis, and progressive skin fibrosis [41]. On the other hand, sarcoidosis is associated with increased IFN-γ-inducible chemokines, CXCL9 and CXCL10, which correlate with severity in lung function as measured by DLCO and PFT [42]. These chemokines may be a target for therapeutics in the future.

As there are potentially similar immunopathologic pathways in sarcoidosis and systemic sclerosis, further research on evaluation and management strategies may shed light on improving patient outcomes. We additionally encourage clinicians to engage in a detailed investigation into potential overlapping lung disease in the presence of discordant CT and pathologic findings.
